# Phenotypic Resistance and the Dynamics of Bacterial Escape from Phage Control

**DOI:** 10.1371/journal.pone.0094690

**Published:** 2014-04-17

**Authors:** James J. Bull, Christina Skovgaard Vegge, Matthew Schmerer, Waqas Nasir Chaudhry, Bruce R. Levin

**Affiliations:** 1 The Institute for Cellular and Molecular Biology, The University of Texas, Austin, Texas, United States of America; 2 Center for Computational Biology and Bioinformatics, The University of Texas, Austin, Texas, United States of America; 3 Department of Integrative Biology, The University of Texas, Austin, Texas, United States of America; 4 Department of Veterinary Disease Biology, University of Copenhagen, Frederiksberg, Denmark; 5 Department of Biology, Emory University, Atlanta, Georgia, United States of America; 6 Atta-ur-Rahman School of Applied Biosciences, National University of Sciences and Technology, Islamabad, Pakistan; Leiden University, Netherlands

## Abstract

The canonical view of phage - bacterial interactions in dense, liquid cultures is that the phage will eliminate most of the sensitive cells; genetic resistance will then ascend to restore high bacterial densities. Yet there are various mechanisms by which bacteria may remain sensitive to phages but still attain high densities in their presence – because bacteria enter a transient state of reduced adsorption. Importantly, these mechanisms may be cryptic and inapparent prior to the addition of phage yet result in a rapid rebound of bacterial density after phage are introduced. We describe mathematical models of these processes and suggest how different types of this ‘phenotypic’ resistance may be elucidated. We offer preliminary *in vitro* studies of a previously characterized *E. coli* model system and *Campylobacter jejuni* illustrating apparent phenotypic resistance. As phenotypic resistance may be specific to the receptors used by phages, awareness of its mechanisms may identify ways of improving the choice of phages for therapy. Phenotypic resistance can also explain several enigmas in the ecology of phage-bacterial dynamics. Phenotypic resistance does not preclude the evolution of genetic resistance and may often be an intermediate step to genetic resistance.

## Introduction

Bacterial viruses – bacteriophages or ‘phages’ – were central to the foundations of modern genetics and molecular biology, and their properties have been studied thoroughly. And for well over half a century, the standard model for a lytic phage invading a high-density bacterial population has been one of the evolution of genetic resistance: the phage will kill sensitive bacteria, whereupon either sensitive bacteria will be maintained at low density thereafter, or genetically resistant mutants that are initially rare will ascend to abundance [1]–[4]. If resistance incurs a fitness cost, the final population will include a mix of sensitive and resistant cells, with phage maintained on the sensitive population and possible co-evolution of phage and bacteria [4]–[7]. A dominant dynamical feature of this process is an initial and profound depression of bacterial densities when the phage first invades, with a gradual recovery of bacterial densities if resistant cells were initially present.

We describe a process that has two important differences from the standard model. First, resistance is partial or quantitative, rather than absolute. Second, resistance is phenotypic (environmental, in quantitative genetics phraseology) rather than genetic. When both conditions are satisfied, the bacterial population may respond to phage infection with only a moderate initial drop in density; bacteria recover quickly to a state in which both bacteria and phage are maintained at high density. Phenotypic resistance may be characterized by just one or a few discrete states in the population up to a virtually continuous distribution of degrees of partial resistance.

The ramifications of phenotypic resistance are subtle but potentially profound. Partially resistant bacteria may be absent or at low frequency in the initial population, and thus be cryptic, but they can ascend quickly and have a major effect on dynamics. They may thus be a cause of phage therapy failure, and understanding the phenomenon may avoid such failures. Phenotypic resistance may also explain the stable coexistence of phage in bacteria in environments that are otherwise predicted to experience oscillations (undamped or of increasing magnitude, e.g., [8]).

The goal of this paper is to elaborate this alternative model and describe some of its consequences. The first section presents and reviews empirical results from a couple well characterized systems to motivate the model. Subsequently, using mathematical models, we describe methods to detect phenotypic resistance and consider its implications to the population and evolutionary dynamics of bacteria and lytic phage.

## Results

### 1. Empirical contradictions with the standard model

**Figure 1:**
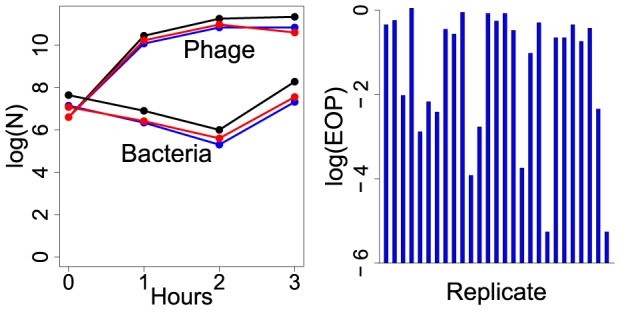
Evidence of phenotypic resistance. (Left) Short-term dynamics of *E. coli* O18:K1:H7 with phage K1-ind(1). This phage does not require the K1 capsule for infection, and although this phage grows well when introduced at low density to a bacterial culture, it and others like it perform poorly in preventing mortality of mice infected with the bacterium [24]–[27]. The assay here grew bacteria 1 hr at 37 in 10 mL LB with aeration, added phage at an MOI of 0.1 (time 0), then plated phage and bacteria from this culture at times shown by dots. Three replicates were performed, each designated with a different color. Cells from the final, 3 hr culture were plated and 5–10 colonies tested for phage sensitivity; most colonies in each replicate were sensitive to the phage [ratios of sensitive to total were (9+1)/10, (6+1)/7, and (5+2)/9, the second number in parentheses indicating intermediate sensitivity]. As the density of phage exceeded that of cells by 3–4 orders of magnitude at the time of plating, we often observed that sensitive colonies were contaminated by low densities of phage, so these numbers should be considered underestimates of sensitivity. (Right) Efficiency of plating (EOP) values of phage F287 on 27 independent colonies of *Campylobacter jejuni* obtained from a 24 hr culture inoculated from a single colony. The culture has already accumulated considerable variation in phage sensitivity in the absence of phage. EOP is the ratio of number of plaques obtained on the isolate divided by the number of plaques obtained on a standard sensitive strain. In both panels, the vertical axis is log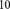 of the respective quantity.

The perspective in this paper is motivated by observations that defy the standard model: in short-term bacterial cultures exposed to phage, an initial decline in bacterial densities caused by phage killing is followed by a bacterial rebound amid an abundance of phage, but the cells are predominantly sensitive. This pattern was reported recently for *Streptococcus thermophilus*, and the anomaly of the rise in sensitive bacteria was noted [9]. We likewise report a similar pattern here for *E. coli* O18:H7:K1 (Fig. 1). The anomaly is that bacterial densities increase after initially being depressed by phage, yet phage density is higher during the bacterial increase than it was during the decrease – constancy of parameters cannot explain this pattern [8], [9].

Various mechanisms could underlie the rise of sensitive bacteria in the presence of phage. The most plausible is a decline in the effective adsorption rate of phage to bacteria, as a low adsorption rate constant is known to enable coexistence of high densities of phage and sensitive bacteria [8], [10]. Any mechanism that involves loss of phage or irreversible binding of phage to debris would reduce viable phage density rather than leave it intact. The fact that the ascending bacteria are sensitive violates the classic model of a genetic resistance mutation. There are several types of non-genetic, partial resistance (low adsorption rate constant) that could explain it, however, as considered next.

### 2. Interpreting and modeling phenotypic resistance

#### 2.A. Importance of the adsorption rate in phage-bacterial dynamics

In well mixed cultures, phage-bacterial interactions are modeled as a mass action process [1], [2]. The rate at which phage-bacterial collisions occur and result in infection is represented as the product of bacterial density (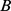, per mL), phage density (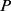, per mL) and the adsorption rate constant (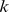, mL per min): 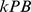. The adsorption rate constant is a combined rate at which a bacterium and phage encounter each other by diffusion and also in which the encounter results in an infection. It is typically bounded at the high end by a value between 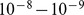 [1], but values 2–3 orders of magnitude lower can be observed even when phage are maintained [8]. The fact that the adsorption rate constant is bounded at the high end by such a tiny value is due to physical constraints: the small sizes of bacteria and phage greatly limit their chance encounter from diffusion in a mL of liquid [1]. Thus, when the adsorption rate constant lies near this upper boundary, virtually all collisions that do occur between phage and bacteria result in infection – the probability of infection per contact is near 1. Smaller values of 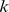 thus reflect reduced probabilities of infection per contact.

It is well understood that, in a flow-through system (e.g., chemostat) with a single type of phage and bacterium, the adsorption rate has a major effect on equilibrium densities. The equilibrium densities are in fact inversely proportional to the adsorption rate constant [8]. Furthermore, low adsorption rates (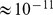 or less) lead to the paradoxical outcome that both phage and sensitive bacteria coexist at high densities. That result is key to understanding the importance of quantitative variation in adsorption rates because the individual bacteria with low adsorption rates differentially survive phage attack and convert the bacterial population into a state of predominantly low adsorption rate.

#### 2.B. Three possible mechanisms of phenotypic variation in resistance

Within the range of attainable values, the adsorption rate constant is a property of both the bacterium and phage, affected by genetic and by non-genetic properties of each. Changes in the number of receptors on the bacterium will affect the adsorption rate constant as will variation in the number of tail fibers on the phage. Bacteria can completely escape phage infection by modification or elimination of essential receptors, but they can also quantitatively reduce the probability of infection by reducing or masking receptors. Indeed, several types of variation in bacterial resistance to a phage can have non-genetic causes. It has long been appreciated that starved or stationary phase cells can have low adsorption rates, perhaps because of reduced cell size and also because of reduced receptor abundance [11], [12]. Even with actively growing cells, any factor altering the abundance or availability of receptors can impart quantitative levels of resistance. It is thus plausible that populations of genetically uniform bacteria contain a mix of cells that collectively present a distribution of different adsorption rates (e.g., Fig. 1).

At least three mechanisms of phenotypic resistance can be envisioned. We acknowledge that most of these mechanisms are speculative, being inferred indirectly from other types of evidence on bacterial phenotypes:

- Induced. Reduced adsorption is due to a change in uninfected bacterial gene expression after exposure to products of phage-lysed bacteria in the environment.
- Intrinsic. Reduced adsorption is due to a physiological or gene expression state that exists in a subset of the population prior to the introduction of phage. It is a consequence of normal environmental variation or a high mutation rate (Fig. 1).
- Dynamic. Reduced adsorption results from degradation or blocking of bacterial receptors by phage proteins released by lysed cells [11].

A category whose effects overlap intrinsic resistance is phase variation. Phase variation is a genetic process of rapid mutation between states of expression and suppression of genes encoding bacterial surface receptors, and the consequences of phase variation to evolution of resistance to phages are well appreciated [13], although the implications to dynamics have not been addressed. A major difference between phase variation and our use of intrinsic variation lies in the degree of resistance of individual cells. With phase variation, cells in the resistant state may be completely resistant (lacking the receptor), whereas with intrinsic variation, the resistant state is partial – cells can be infected. Thus bacteria with intrinsic resistance would manifest clear plaques but phase variation would manifest turbid plaques.

We offer these categories as possibilities. Although there seems to be appreciation among phage biologists of some of these mechanisms, for the most part, they and their consequences have not been systematically explored. Nor is it necessarily obvious how to recognize and distinguish them. We thus work out their consequences below.

### 3. Bacterial populations prior to phage invasion

We offer models to illustrate several consequences of quantitative, phenotypic resistance. The category of ‘intrinsic’ variation is distinct from the other two categories in that the reduced adsorption rate exists prior to exposure of the population to phages. Because it exists prior to phage invasion, intrinsic variation can be assessed in phage-free cultures, rendering its properties both subtle but easy to model. Thus more of our treatment is on intrinsic variation than on the other two mechanisms. Although most derivations assume just 2 bacterial types in the population, we expect that phenotypic resistance may involve a broad spectrum of partial resistance levels (e.g., Fig. 1).

#### 3.A. Abundance of different types in a phage-free bacterial population

Consider the bacterial population before phage are introduced. Phenotypic resistance in the absence of phage can be ignored with induced and dynamic mechanisms of phenotypic resistance, but it will be present under the intrinsic mechanism. If there are two bacterial states (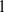, 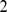) that have no effect on reproduction and survival in the absence of phage, dynamics of the two cell types obey
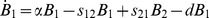


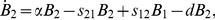
(1)where 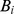 represents the density of bacterial type 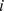, 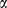 is the growth rate, 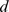 the death rate, and 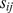 is the switching rate from state 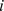 to 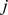. A superior dot indicates differentiation with respect to time.

Without loss of generality, we assume that the death rate matches the birth rate, which will be true at equilibrium under a diversity of mechanisms. The equilibrium abundance of the two states is thus given by
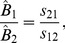
(2)with a hat indicating equilibrium. Thus the higher the relative switching rate away from a state, the lower the state's equilibrium relative abundance. The simplicity of this result is due to our assumption that the two states are the same in all parameters except switching rates.

With more than two states and arbitrary switching, 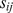, there is no straightforward analytical solution. Equilibrium satisfies
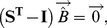
(3)where 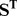 is the transpose of matrix 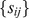, 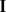 is the identity matrix, and 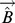 is the vector of equilibrium bacterial densities. The matrix 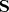 has the property that row elements sum to 1. We have not explored the possibility of an analytical solution to (3) nor can we suggest that a solution is unique. If the 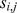 are known, however, a numerical solution is straightforward.

#### 3.B. Survival of bacteria exposed to high densities of phage: differences in adsorption rate are profoundly important

Under the intrinsic mechanism, many adsorption rate states may initially be rare in the population. Here we show that the addition of a high concentration of phage to the bacteria results in large and rapid changes in the relative abundances of different cell types. These rapid changes may be useful in detecting the otherwise cryptic variation in adsorption rate.

It is first noteworthy that standard adsorption rate assays will be insensitive to minor bacterial variants in the population. Adsorption rate assays are measured from the decline of free phage density in a culture with bacteria – phage are added at time 0, and the number of attached versus unattached phages are counted at one or more later times. To understand how the measured adsorption rate is affected by variation among bacteria, we need to know how the free phage density declines as a property of the different bacterial states in the population. Let the density of bacterial type 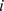 be 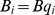, where 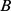 is total bacterial density, 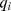 is 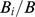, hence 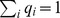. The rate at which phage are lost to adsorption is
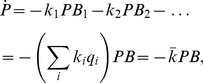
(4)where 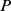 is phage density, 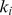 is the adsorption rate constant for bacterial type 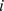, and 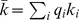. These dynamics assume the absence of phage reproduction, as applies in adsorption rate assays. And if bacteria already adsorbed to phage continue to adsorb additional phage, the densities of bacteria adsorbing phage can be assumed to stay constant because the sum of live and previously infected bacteria does not change (provided the time frame is confined to the interval before infected bacteria lyse – burst open). From this result it is apparent that any rare bacterial types – those with low 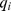 – will have little effect on the measured adsorption rate, which is an average over all types.

Now consider changes in densities of surviving bacteria as a function of their adsorption rates. Let 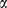 be the growth rate of the uninfected bacteria. Assuming two types of cells, parameters as above, and no phage reproduction (e.g., a brief interval after phage are added to the culture), the rates of change of surviving bacteria in the presence of phage are
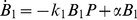
(5)

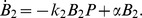



To get an approximate and easily interpretable sense of dynamics, first assume constant phage density (and again no phage reproduction). The density over time of uninfected bacteria of type 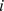 (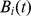) follows
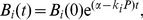
(6)so the ratio of two surviving bacterial types obeys
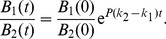
(7)When phage density is high (on the order of the inverse of the larger adsorption rate constant), even a ten-fold difference in adsorption rate has a profound effect on relative abundances of surviving bacterial types in as little as 10 minutes.

This result points to a way of detecting standing variation in adsorption rate under the intrinsic model: infect a bacterial culture with a high density of phage and plate to measure bacterial survival before phage reproduce (which is also before bacteria lyse). Different cell types will survive at different rates. A more exact calculation than that in (7) is required because the density of free phage is not constant but declines as phage adsorb; equations from Bull and Regoes [14] can be used to correct for this effect. Returning to eqn (4) but making explicit the constancy of bacterial densities for adsorption,
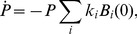
(8)with the solution
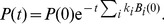
(9)Note that bacterial densities are held constant for the sake of phage adsorption, even though some or many of those bacteria are infected and will be unable to form colonies. We are, however, also interested in bacterial survivors, but we need this result to make that calculation.

The decay in each type of surviving (uninfected) bacteria can now be calculated. Assuming a time interval so short that bacterial reproduction can be neglected, eqns (5) and (9) yield
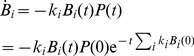
(10)

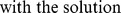


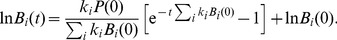
(11)


**Figure 2:**
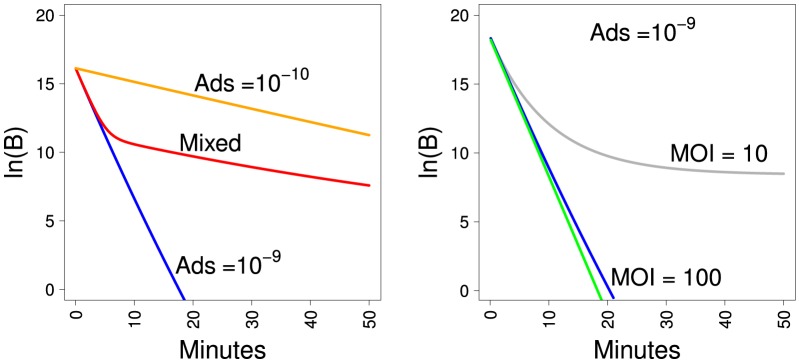
Survival curves of bacteria. Bacteria are exposed to a high initial density of phage, and survivors (uninfected) measured before burst occurs; cells continue adsorbing to phage regardless of how many phage have already adsorbed. The vertical axis represents the natural log of surviving bacterial densities. (Left) Survival curves for homogeneous bacterial populations. The blue curve represents a 100-fold excess of phage to cells (MOI is multiplicity of infection, or the ratio of phage to cells), whereas the gray curve is for only a 10-fold excess; initial phage densities are 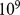/mL, adsorption rate constants are 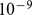 mL/min. The blue and gray curves account for a decay in phage concentration as phage continue adsorbing to bacteria. The green curve is strictly linear for comparison to the blue curve. The plateau of the gray curve is due to nearly all phage having adsorbed after 20–30 min. Note that all three curves are adjusted to start at the same bacterial density for visual comparison of slopes, even though the blue curve should start at a 10-fold lower bacterial density than the gray curve. (Right) Survival curves for two homogenous bacterial populations differing in adsorption rate constants (orange: 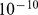 mL/min; blue: 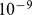 mL/min). The red curve is for a mix of 99% cells with a rate constant of 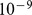 and 1% cells with rate constant 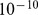. Thus, despite the small fraction of cells with low adsorption rate in the mixed population, there is a profound effect on the survival curve after just a few minutes of exposure. Initial phage density is 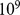/mL, and cells are at 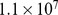/mL for each homogenous population and 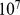/mL and 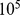/mL for the two types in the mixed population.

Bacterial survival curves are shown in Fig. 2; it is assumed that the only effect on phage density is loss through adsorption, hence no release of phage progeny occurs within the plotted time frame. The left panel assumes a homogenous cell population (a single 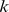 for all cells) in which free phage initially outnumber bacteria. The green and blue curves are for 100-fold excess of phage; the green curve is forced to be linear, as would be obtained with strictly constant phage density, whereas the blue curve accounts for the declining phage density due to adsorptions; curvature of the blue deviates almost imperceptibly from linear. The gray curve represents a 10-fold excess of phage and violates linearity profoundly; flattening of the curve is due to the loss of nearly all free phage from adsorption after 20–30 min.

The right panel compares two curves for pure cultures with one curve for a culture containing a mix of bacteria with two resistance levels. Phage start with a 100-fold excess over cells; the blue curve assumes that all cells have an adsorption rate of 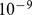, the orange curve assumes that all cells have an adsorption rate of 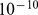; the red curve assumes that 99% of the cells have an adsorption rate of 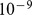 and the other 1% have an adsorption rate of 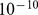. Thus a small fraction of cells with reduced adsorption can have a major effect on the survival curve when there is a large excess of phage. It is thus evident that this method offers a means of detecting standing variation in adsorption rate.

This survival curve will be sensitive to the nature of phenotypic resistance and to the properties of the phage suspension added to the cells. The foregoing calculations assumed that resistance is pre-existing (intrinsic) and that no induction of resistance or dynamic resistance occurs. Depending on the time course of the bacterial response, qualitatively similar shapes – curve flattening – would be obtained if the bacteria had no pre-existing variation in resistance but exhibited dynamic or induced resistance in response to the addition of phage. Use of a purified phage preparation (e.g., from a cesium gradient) would presumably avoid both induced and dynamic resistance prior to lysis. Conversely, pre-exposure of the bacteria to a lysate in which all the phage were killed (e.g., by heat or radiation) might cause a marked shift in the adsorption rate before viable phage were added and thus reveal dynamic or induced resistance.

Empirical determination of the survival curve is not trivial. To use these equations, plating of surviving bacteria must be done before burst – because we do not allow phage reproduction or reduction in cells adsorbing phage – but infected bacteria will burst after plating. Once the density of surviving bacteria has fallen several logs, larger volumes must be plated and the plates will have a large excess of phage that may kill otherwise surviving microcolonies; use of phage anti-sera or specific detergents may reduce the effect. In addition, survival rates may be biased upward because of anomalies such as clumps and debris protecting bacteria from phage. Empirical determination of the survival curve will be least problematic when the variation in adsorption rate is evident over only a few logs drop in viable cell density, while volumes plated are still small.

### 4. Phage growth

#### 4.A. Phage invasion is determined by the average adsorption rate, so phenotypic resistance is cryptic by this measure

As a first step toward understanding the impact of phenotypic resistance on phage dynamics, we consider phage invasion – is phenotypic resistance readily detectable when a phage first invades? Phage growth is easily quantified, and if this measure of invasion reflects long term dynamics, it would provide a convenient short-cut to more elaborate assays. Invasion of a phage into the bacterial population is modeled as if viable bacteria are not limiting and maintain a constant density, in which case exponential phage growth applies soon after the introduction. Following Campbell [2], the rate of change in phage density (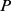) in a population with a single cell type (single adsorption rate) is given by
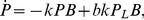
(12)where 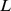 is the lysis time, 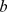 is burst size, and 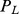 is the density of phage 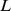 time units in the past. Bacterial density 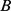 is assumed constant in time, and we ignore phage loss from death and washout (which is easily included).

The model is readily expanded to include multiple bacterial types that have different effects on phage life history. As above, let 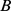 denote total cell density and 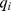 be the fraction of bacteria with adsorption rate 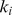, with lysis time 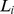 and burst size 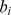. With constancy of total bacterial density as well as of the 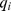, equation (12) becomes
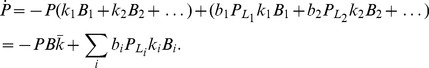
(13)When exponential growth has been attained (requiring a stable age-of-infection distribution, [15]), the phage growth rate 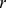 obeys
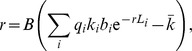
or if all infections have the same lysis time and burst size,
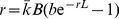
(14)Thus if adsorption rate is the only variable, invasion proceeds according to the average adsorption rate in the population, a result that might be inferred from (4) above. Bacteria whose abundance is at most a few per cent have little or no perceptible impact on how rapidly the phage population expands at low density, so their ultimate impact on dynamics is cryptic at invasion. The model is general to all three mechanisms of phenotypic resistance, in that phage are rare enough during invasion that the induced and dynamic mechanisms will not operate.

#### 4.B. Full dynamics with intrinsic resistance

To evaluate the dynamics after invasion, we use a differential equation model similar to that in Levin et al. [8]. There are 6 variables: sensitive bacteria (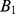), semi-resistant bacteria (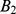), infected bacteria of each type (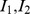), phage (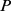), and resource level in the environment (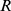). The two types of bacteria accommodate our definition of intrinsic resistance, although the model does not distinguish genetic and non-genetic bases for the two types, provided the switching rates apply. Loss and gain terms for bacteria are straightforward; infected bacteria are included because they continue to adsorb phage and use resources up to lysis. Gains to phage density come solely from the lysing of infected bacteria, whereas phage losses include adsorption to infected and uninfected bacteria as well as death/washout (which diminishes all variables at the same per capita rate). Also, the bacterial growth rate declines as bacterial abundance increases; we use a standard (if detailed) model with a Monod function of metabolism to approximate biological reality [8] but suggest that many simpler functions would behave similarly.

Our equations follow:
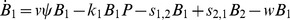
(15)

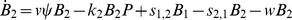


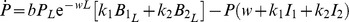


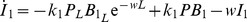


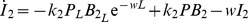


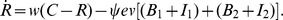
Parameters and variables are defined in Table 1. A subscript 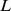 indicates the value of the variable 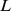 minutes in the past – the time from infection to lysis of the bacterium.

**Table 1:** Variables and parameters.

Notation	Description	Values
Variables		
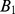	density of sensitive bacteria	
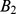	density of partially resistant bacteria	
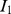	density of infected bacteria of type 1	
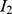	density of infected bacteria of type 2	
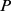	phage density	
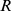	resource level	
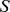	density of substance affecting adsorption in dynamic model	
Functions of variables		
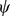	Resource scaling level ( 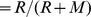 )	
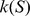	Dynamic adsorption rate ( 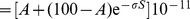 )	
Parameters		
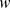	washout/death rate	0.1
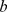	burst size	200
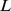	lysis time	25
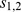	transition rate from sensitivity to partial resistance	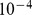
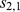	transition rate from partial resistance to sensitivity	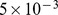
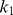	adsorption rate constant for 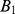	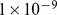
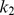	adsorption rate constant for 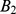	varies
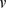	maximum bacterial growth rate	0.35
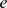	conversion efficiency	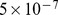
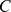	input resource concentration	100
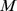	Monod constant	0.25
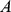	minimum adsorption rate determinant in 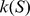	3, 10
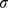	efficacy parameter in 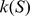	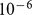 , 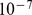

To obtain a sense of model behavior, four runs of different conditions are illustrated (Fig. 3). The upper left panel shows the behavior for a baseline set of parameters and initial conditions, and the other three panels change one of those values or conditions. In the absence of phage, semi-resistant bacteria (red curve) would be maintained at 2% of the population. With phage present, those bacteria are invariably maintained at a higher density than the sensitive cells. The various results indicate the sensitivity of outcomes to small changes in parameter values.

**Figure 3:**
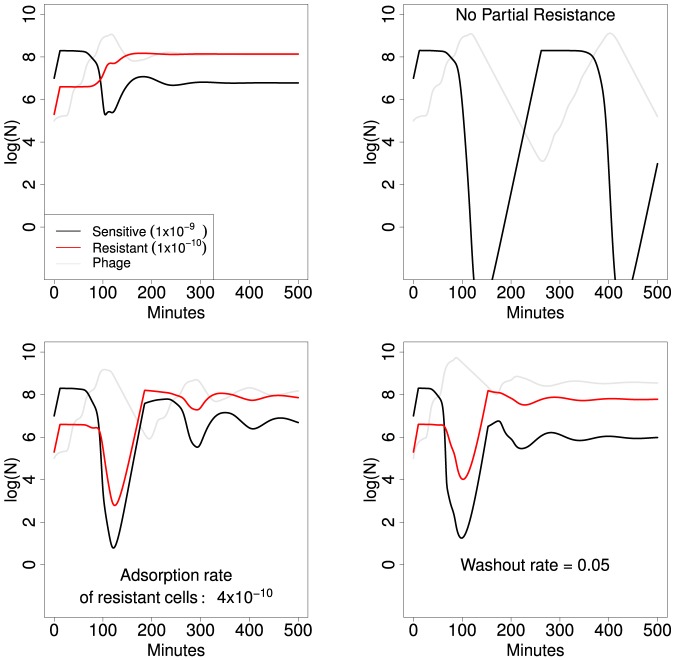
Iterations of the ‘intrinsic’ model, as given by eqn(15). The upper left panel represents a run with baseline parameter values (Table 1; the key gives adsorption rate constants), and only those properties differing from this baseline are noted in the other panels. The three other trials differ in one respect from the upper left: partially resistant bacteria are absent (upper right), the adsorption rate constant of the partially resistant bacteria is increased 4-fold (lower left), or the washout rate is halved (lower right). The red curve represents density of the partially resistant cell type, black is of the sensitive type, and light gray is of phage. In the absence of phage, density of the partially resistant bacteria would be 2% of the total, but partially resistant bacteria always comprise the majority when phage are present (the upper right panel is a control run in which partially resistant bacteria are absent, illustrating the typical oscillations). Initial densities were 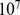 for 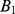, 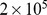 for 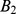, 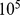 for P, 100 for 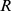, and 0 for both types of infected cells. The vertical axis is log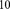 of the respective density.

Oscillations are typical in this type of model [8], but even with oscillations present, semi-resistant bacteria often remain at high density, usually one to two orders of magnitude above sensitive cell density. Thus phage profoundly change the relative abundances of different resistance levels, and phage are less effective at controlling semi-resistant cells.

#### 4.C. Full dynamics with induced or dynamic resistance

Our model of dynamic resistance differs from that of intrinsic resistance in three ways: (i) only a single bacterial type is present, (ii) the variable 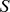 is introduced for the concentration of a substance released by lysing cells, and (iii) adsorption rate is a declining function of 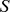, 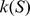. Equations are much the same as in the previous model except for the effect of 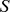 and the omission of a second bacterial type:
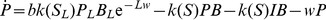
(16)

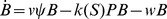


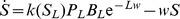


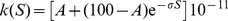


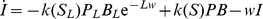


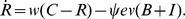

Table 1 again provides definitions and values, except that subscripts for 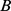 and 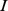 are omitted in this model. The adsorption rate function 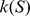 ranges from a maximum of 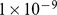 when 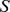 is 0 to a lower bound of 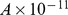 (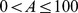). The impact of 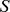 between these extremes depends exponentially on the product 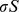, so 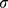 can be regarded as an efficacy parameter (baseline 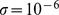). These equations can represent either dynamic resistance (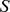 interferes with adsorption) or induced resistance (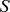 causes bacteria to reduce receptor levels), although the effect of induced resistance might be more accurately modeled as a delay function.

**Figure 4:**
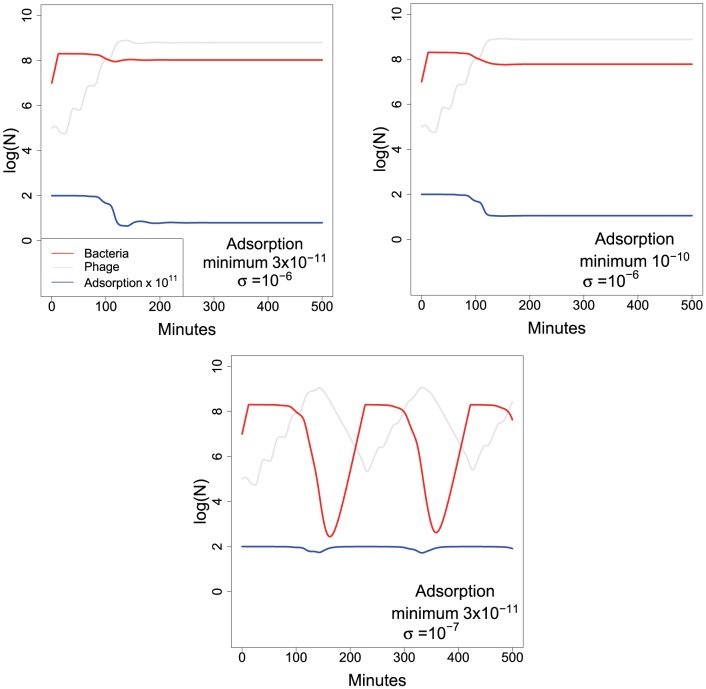
Iterations of the ‘dynamic’ or ‘induced’ model of phenotypic resistance, as given by eqn(16). Only one bacterial type is present. The maximum adsorption rate is 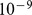 in all three panels, but the minimum adsorption rate is 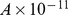: 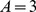 in the upper left and lower panels, 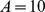 in the upper right panel. Collectively these figures illustrate that the magnitude of oscillations is moderately robust to differences in the minimum absorption rate but sensitive to the adsorption rate function (as determined by 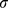). Red (light gray) curves are for bacteria (phage), the blue curve is the adsorption rate multiplied by 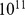, which varies with 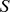. Parameter values are as given in Table 1 except where indicated. Initial densities were 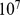 for 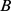, 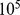 for P, 100 for 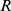, 100 for 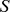, and 0 for infected cells. The vertical axis is log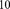 of the respective density.

Fig. 4 shows that dynamic/induced resistance can stabilize oscillations. As expected, the stabilizing effect depends on how low the adsorption rate constant becomes through the impact of 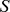. By comparison to the first two panels, the third panel shows that the dynamics are highly sensitive to the ‘efficacy’ parameter 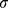.

## Discussion

Bacterial genetics of the 1960s and 1970s was dominated by the model of a one-to-one relationship between genotype and phenotype. Bacteria could or could not ferment specific sugars, could or could not synthesize amino acids or vitamins, and were susceptible or resistant to killing by lytic bacteriophages. In recent years it has become clear that there is qualitative as well as quantitative phenotypic variation among genetically identical members of the same bacterial population. Prominent, extreme examples are the genetically identical states of competence and cannibalism in *Bacillus subtilis* and the phenomenon of ‘persistence’ and its associated phenotypic resistance to antibiotics [16]. More broadly, there is now a wide appreciation of cell-cell variation in metabolism within populations [17]–[19], with relevance to drug resistance [20]. Phase variation in bacteria (e.g., [13]) is an intermediate category that lies between the standard model of genetic resistance and that of phenotypic resistance – its rapid switching between genetic states renders it similar to our use of phenotypic resistance, but the complete resistance and modification of the DNA sequence renders it ‘genetic.’

The existence and relevance of bacterial phenotypic variation to phage dynamics has received little attention. Weitz and Dushoff ([12]) developed a model in which the adsorption rate constant declined with increasing cell density, reflecting the common observation that cells at or near stationary phase do not lead to productive infections [21]. That model has multiple stable dynamical states, with phage unable to invade at high bacterial density but maintained at lower cell density, for example. Closer to the theme of our study, a recent article presented evidence for phenotypic variation in the susceptibility of bacteria to lytic phage [9]: when infected with the phage 2972, a subset of a genetically susceptible population of *Streptococcus thermophilus* not only survives in the presence of high phage densities, it replicates. Here we presented qualitatively similar results for an *E. coli* strain and its lytic phage, reported within-culture variation in efficiency of plating for a *Campylobacter* strain and its phage, and also addressed the dynamical implications of phenotypic resistance.

The rapid replication of bacteria phenotypically resistant to phage distinguishes the phenomenon from bacterial persisters that resist antibiotics – whose cells do not replicate. The replication of phenotypically resistant bacteria enables them to rebound to potentially high densities in the presence of phages, leading to the enigma that initially effective phages cease to control bacteria even though the bacteria technically remain sensitive.

We offered three possible types of phenotypic resistance: induced, intrinsic, and dynamic. Of the three, only intrinsic resistance exists prior to phage introduction. The genetic and molecular mechanisms behind the different types remain largely unexplored, however. Furthermore, mechanisms of intrinsic resistance may span the spectrum from formal genetic mutations to epigenetics to stochastic variation in protein numbers following cell division. Indeed, phase variation is a mechanism of intrinsic resistance that generates rapid switching of resistance states in ways that might appear to be epigenetic or non-genetic. Considerable empirical effort may thus be required to distinguish the alternative mechanisms.

The implications of phenotypic resistance to phages have both practical and basic dimensions. In phage therapy – the use of phages to control medical and industrial bacterial parasites and pests – phenotypic resistance can lead to a mismatch between the short term growth of a phage and its long term ability to control bacterial populations. Thus a phage that grows well when first introduced may transform the bacterial population into a state that is recalcitrant to phage control even though the bacteria remain sensitive. This mismatch can complicate the wise choice of phages suitable for therapy, and an appreciation of how phenotypic resistance arises may aid the choice of phages suited to therapy (see below).

From the broader perspective of phage-bacterial dynamics, phenotypic resistance may explain a long standing enigma – the failure to observe wild and undamped oscillations in chemostats and other experimental populations. The theory for bacterial-phage growth in chemostats has been worked out for over 3 decades [8]. There are straightforward equilibria [2], [8], but simulations reveal a strong tendency for oscillations around those equilibria, and the oscillations do not dampen (unpublished and [8]). In stunning contrast, empirical studies of phages in chemostats have consistently failed to observe such behavior.

Schrag and Mittler [22] addressed this problem and suggested that wall populations in chemostats acted as refuges that stabilized the dynamics. It is now also appreciated that biofilms provide refuges for bacteria while also enabling phage to ‘graze’ upon the planktonic migrants from the biofilm [23]. All three systems – wall growth, biofilms, and phenotypic resistance – give rise to the paradox of high levels of phage maintained on sensitive bacterial populations. Phenotypic resistance is unique in providing a mechanism of escape in purely planktonic bacterial populations and thus may be relevant to phage-bacterial dynamics many contexts.

Phenotypic resistance to phages has not been explored in depth, so little is known about its mechanisms. We can nonetheless suggest how its properties might differ from those of genetic resistance in its implications for the coexistence of phage and bacteria. One is that mechanisms of phenotypic resistance may be more diverse and abundant than mechanisms of genetic resistance because they can be affected by subtle changes in gene expression and by molecules in the external environment. Second, and for similar reasons, phenotypic resistance may have less severe fitness consequences than does genetic resistance. Last, as noted above, phenotypic resistance will often be cryptic in the sense that the initial, phage-free population gives little evidence of it, yet it rapidly dominates the population when phage are added. The properties of phenotypic resistance may be specific to the receptors used by a particular phage and thus differ for different phages; genetic resistance also exhibits dependence on phage receptors, of course.

The perspective here may solve a puzzle in the classic phage therapy work of Smith and Huggins [24]. They showed that, when treating an artificial *E. coli* O18:H7:K1 infection of mice, phages requiring the K1 capsule for infection (K1-dep) rescued at nearly 100%, but phages whose infection was independent of the capsule (K1-ind) were much less successful. Although Smith and Huggins commented that K1-ind phages grew poorly and often failed to clear a culture, Bull et al. [25]–[27] found that the growth rate of K1-ind phages was high both in culture and in mice, certainly more than enough to overwhelm the bacterial population if extrapolated across a few hours. It now appears relevant that the growth rate assays of Bull et al. [25], [26] were conducted at low MOI and thus would not be affected by phenotypic resistance (recall eqn(14)), whereas the alternative outcomes of lysis versus non-lysis of an entire culture would be affected by phenotypic resistance. By extension to other systems, phenotypic resistance could be a cause of phage therapy failure even though the phages are able to form clear plaques and grow well when first introduced to an infection or culture.

If phenotypic resistance explains the failure of K1-ind phages to cure an infection or clear a culture, is there a plausible reason that phenotypic resistance is not a problem for K1-dep phages in the Smith-Huggins system? Bull et al. [26] noted that K1-dependent phages from different taxonomic groups all encoded endosialidase tailspikes. In infections of mice, free enzyme produced as unattached tailspikes will contribute to infection clearance by stripping capsules and thereby augmenting the immune system (e.g., treatment with enzyme alone cures the infection, [28]). This mechanism should not contribute to clearing an *in vitro* culture however, as there is no immune system to remove cells with reduced capsules. Instead, enhanced clearing of a culture might be attributable to limited phenotypic resistance, if complete loss of the capsule through phenotypic means is rare. At present, this model is conjecture but is readily testable.

If phenotypic resistance is sufficiently prevalent as to commonly hamper therapeutic success of arbitrarily chosen phages, a fruitful avenue of research will be to look for generalities in the foundations of phenotypic resistance. Some types of phage receptors may be less prone to the problem than others, and if the rules of phenotypic resistance can be discovered, it may be possible to screen phages and choose ones least likely to fail in the application. The work with *E. coli* O18:H7:K1 is one possible example of the feasibility of this approach, although in that system, there may be a dual benefit *in vivo* of phages that target the bacterial capsule.

Phenotypic resistance does not prevent the evolution of classical genetic resistance. Genetic resistance may still be favored in the long term because it reduces the death rate from phage, although phenotypic resistance should ascend first and then slow the ascent of genetic resistance, and observations restricted to the long term will not easily detect phenotypic resistance after it has been replaced by genetic resistance. However, the dynamical behavior of the phage and bacterial populations with a mix of genetic and phenotypic resistance may nonetheless exhibit signatures characteristic of phenotypic resistance (in preparation).

## Materials and Methods

### Bacteria and phage

*E. coli* O18:H7:K1 strain RS218 from frozen aliquots was added to 10 mL LB broth (10 g NaCl, 10 g Bacto tryptone, 5 g Bacto yeast extract per liter) in 125 mL flasks and grown at 37

C with agitation for 1 hr to a density of 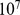/mL. Phage K1-ind(1) [26] were added to a tenth the bacterial concentration and the culture grown without dilution for another 3 hr. Phage and bacteria were sampled at hourly intervals. Bacteria were plated in soft agar (7 g Bacto agar/L in LB) overlaid on agar plates (15 g Bacto agar/L in LB) and incubated overnight. Plating bacteria in soft agar maintains small colony size and thus reduces the contact rate with phage also present in the sample.

Bacterial sensitivity to phage was tested on colony isolates by first placing a streak of phage (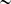
50 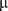
L of a suspension of at least 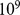 phage/mL) across an agar plate and allowing it to dry. Bacterial colonies were individually suspended in 100 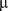
L of LB, vortexed, and 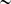
10 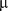
L streaked across and perpendicular to the phage streak. Streaks were scored after overnight growth; sensitive colonies showed dense growth up to the phage but not beyond.

*Campylobacter jejuni* NCTC1168 [29] was routinely cultivated on blood agar base II (Oxoid) supplemented with 5% bovine blood and incubated at 37

C under microaerobic conditions (6% O2, 6% CO2, 4% H2, 84% N2). A single colony was restreaked twice and applied as inoculum for a 40 ml culture in brain heart infusion broth supplemented with 1 mM CaCl2 and 10 mM MgCl2. Cell density was measured by plating at inoculation (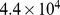 CFU/mL) and at 24 h (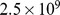 CFU/mL), with plates grown at 37

C under microaerobic conditions as described above. To investigate variation of phage susceptibility within the culture, individual colonies plated at time 0 h and 24 h were restreaked and their distinct phage susceptibility tested by plaque assays with phage F287 [30] essentially as described [31]. Efficiency of plating was calculated relative to the phage titer of the inoculum and measured across a series of dilutions, with 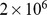 pfu at the highest concentration.

### Simulations and graphics

Differential equations were evaluated numerically in the program Berkeley Madonna (v. 9.0.112 beta) with a step size of 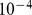 and method Runge-Kutta 4; models will be made available on request. The numerical output was transferred to R for presentation. Figures were drawn in R [32].
